# Fractalkine/CX3CR1-Dependent Modulation of Synaptic and Network Plasticity in Health and Disease

**DOI:** 10.1155/2023/4637073

**Published:** 2023-01-04

**Authors:** N. P. Camacho-Hernández, F. Peña-Ortega

**Affiliations:** Departamento de Neurobiología del Desarrollo y Neurofisiología, Instituto de Neurobiología, UNAM-Campus Juriquilla, Mexico

## Abstract

CX3CR1 is a G protein-coupled receptor that is expressed exclusively by microglia within the brain parenchyma. The only known physiological CX3CR1 ligand is the chemokine fractalkine (FKN), which is constitutively expressed in neuronal cell membranes and tonically released by them. Through its key role in microglia-neuron communication, the FKN/CX3CR1 axis regulates microglial state, neuronal survival, synaptic plasticity, and a variety of synaptic functions, as well as neuronal excitability via cytokine release modulation, chemotaxis, and phagocytosis. Thus, the absence of CX3CR1 or any failure in the FKN/CX3CR1 axis has been linked to alterations in different brain functions, including changes in synaptic and network plasticity in structures such as the hippocampus, cortex, brainstem, and spinal cord. Since synaptic plasticity is a basic phenomenon in neural circuit integration and adjustment, here, we will review its modulation by the FKN/CX3CR1 axis in diverse brain circuits and its impact on brain function and adaptation in health and disease.

## 1. Introduction

**Figure 1:**
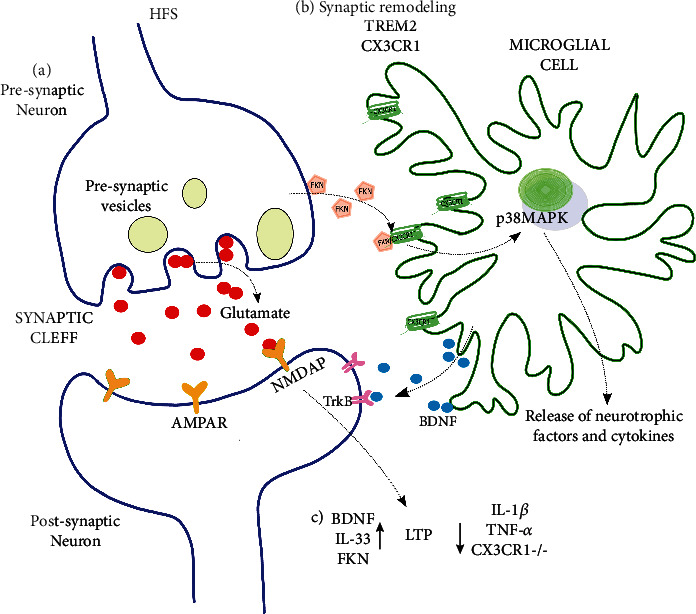
Microglia regulate hippocampal LTP through FXN/CX3CR1 signaling. (a) After high-frequency stimulation (HFS), increased glutamate release overactivates AMPAR but mostly NMDAR, inducing hippocampal long-term potentiation (LTP). (b) Microglia communicate with neurons through CX3CR1, inducing synapse remodeling and promoting plasticity. The lack of CX3CR1 impairs synaptic remodeling and engulfment and spine formation. (c) After HFS, neurons release FKN to activate microglial CX3CR1, leading to the synthesis and release of neurotrophic factors (BDNF) and cytokines, which can enhance (↑) or inhibit (↓) hippocampal LTP. The lack of CX3CR1 or FKN inhibits LTP, which is related to the exacerbated release of IL-1*β* and TNF-*α*.

**Figure 2:**
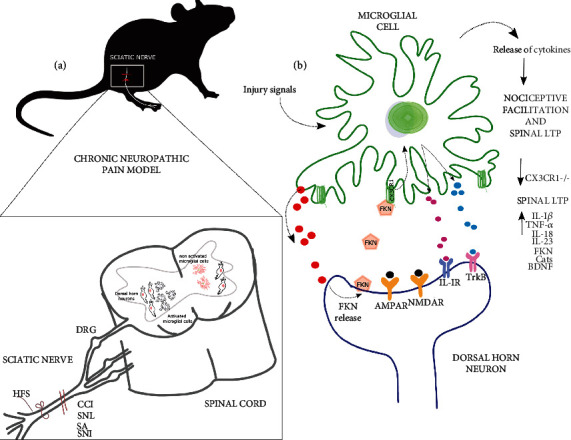
Neuropathic pain and spinal plasticity are modulated by microglial CX3CR1. (a) Neuropathic pain has been studied using several models including nerve high-frequency stimulation (HFS), chronic constriction injury (CCI), sciatic nerve ligation (SNL), sciatic axotomy (SA), and sciatic nerve injury (SNI). These models converge in the activation of spinal microglia. (b) Microglial activation leads to the release of cathepsin S (CatS), which induces the release of fractalkine (FKN) from dorsal horn neurons and its binding to microglial CX3CR1. The activation of CX3CR1 recruits p38 MAP kinase (p38MAPK), which is involved in the release of cytokines and neurotrophic factors that participate in the induction and maintenance of spinal long-term potentiation (LTP) and/or nociceptive facilitation. DRG: dorsal root ganglion; AMPAR and NMDAR: glutamate receptors; IL: interleukin; BDNF: brain-derived neurotrophic factor; TNF-*α*: tumor necrosis factor alpha.

CX3C chemokine receptor 1 (CX3CR1) is a seven-transmembrane domain G protein-coupled receptor involved in neuron-microglia crosstalk within the central nervous system (CNS) [1–7] and leukocytic cell adhesion and migration in the periphery [8]. In the CNS, CX3CR1 is constitutively expressed by microglia in rats [2, 4–7, 9, 10], mice [3, 10, 11], and humans [12–14] (Figures 1 and 2). Although this receptor can be infrequently found in neurons [4, 10, 15] and astrocytes [2], its expression in these cell types is induced during alterations in the brain microenvironment [15] or artificial conditions during cell culture [2, 4, 16]. Within the brain parenchyma, the CX3CR1 expression is constitutively and exclusively expressed by microglia [9, 11–13] (Figures 1 and 2). This expression of CX3CR1 corresponds temporally and spatially with the chemotactic cytokine CX3CL1 or fractalkine (FKN) [3, 4, 8, 17] (Figures 1 and 2), which is exclusively produced by neurons [18, 19] (Figures 1 and 2).

There are two isoforms of FKN, one is soluble and the other is anchored to the membrane [20, 21] (Figures 1 and 2). The membrane-anchored FKN isoform has a 76-amino acid (aa)-long N-terminal extracellular domain, followed by a 241 aa-long mucin-like stalk that connects to an 18 aa-long hydrophobic transmembrane domain that ends with the 37 aa-long intracellular C-terminal [21–25]. The extracellular domain of the membrane-anchored FKN can be cleaved, releasing soluble FKN (Figures 1 and 2) by disintegrin and metalloproteases ADAM 10 [26] and ADAM 17 [27] and by lysosomal cysteine proteases and cathepsin S (CatS) [28, 29]. In either its membrane-anchored or soluble forms, FKN binds exclusively to CX3CR1 (Figures 1 and 2), initiating a cell signaling cascade that modulates microglial function [5, 13, 30–32] (Figures 1 and 2). Of note, FKN can trigger both anti-inflammatory and proinflammatory responses in microglia depending on its concentration, solubility, and peptide size, as well as on microglial age and the neural circuit microenvironment [21, 33–35]. In the next section, the mechanisms by which FKN/CX3CR1 signaling modulates microglia function in different contexts will be discussed.

## 2. Fractalkine/CX3CR1-Dependent Microglial Modulation in Health and Disease

### 2.1. Physiological CX3CR1-Dependent Microglial Modulation

CX3CR1 modulates microglial function in a complex fashion and in a state-dependent manner influencing neuronal survival and excitability, synaptic transmission and plasticity, and thus a variety of behaviors [2, 8, 11, 13, 17]. In physiological conditions, neurons express FKN on the cell membrane and constantly release FKN to the microenvironment, where it can be detected by microglial CX3CR1 [3, 7] (Figures 1 and 2). CX3CR1 activation is normally involved in maintaining microglial cells in a quiescent state [36–39], but also inducing their motility and migration [40, 41], promoting neuronal function stability [42–45], and even acting as a neuroprotective system [18, 19, 37, 46, 47]. However, FKN can also activate microglia [21] and lead to neurotoxic effects [48, 49]. These contradictory effects of FKN/CX3CR1 axis activation may be due to multiple factors: (1) the brain region studied, (2) the experimental conditions in which it is studied, and/or (3) the concentration of pharmacological tool used (endogenous induction or exogenous application also seems to be relevant) [18, 21, 36, 50, 51]. Another source of differences in CX3CR1 or FKN expression or function is the combination of sex and brain structure [21, 52]. For instance, in a stress model induced by intense light stimulation (three hours a day for ten days), the expression of CX3CR1 in the cortex and amygdala is increased in females but decreased in males. Additionally, in the cortex, the same stressful manipulation induces an increase in FKN levels in females but not in males. In contrast, in the hippocampus, FKN levels increased in females but decreased in males [52]. An additional source of differences in FKN/CX3CR1 axis actions is age: young and aged microglia respond similarly to lipopolysaccharide (LPS), but the response to FKN changes during aging [21]. Finally, another source of divergent effect of the FKN/CX3CR1 axis is the neural circuit microenvironment, as FKN can be neuroprotective in control mice [46] and contributes to cell damage in animals lacking FKN, in otherwise identical experimental conditions [46]. These are clear examples of how even in the same model both FKN and CX3CR1 expression and function can vary in structure-, age-, and sex-dependent manners.

Beyond its involvement in the CNS response to challenging conditions, which will be reviewed later, the FKN/CX3CR1 axis plays a major role in healthy brain function, including proper CNS development, by modulating neural network sculpting and maturation [53, 54]. For instance, the FKN expression is increased in specific locations during critical periods of network wiring [53–55], where it regulates CX3CR1-dependent microglial recruitment and their precise location [53–57], promoting neuronal connectivity [54, 55, 58], functional maturation of synaptic contacts [55, 59], neuronal survival of developing neurons, axon outgrowth control, and laminar positioning of interneuron subsets [53, 56, 60–62]. The relevance of this function is revealed when the FKN/CX3CR1 axis is disturbed resulting in abnormal CNS development [53, 60, 61]. For example, the absence of CX3CR1 leads to delayed synaptic maturation in the auditory brainstem, affecting the latencies of the auditory brainstem responses [59]. Similarly, CX3CR1 deficiency impairs the functional maturation of thalamocortical synapses in the barrel cortex [55], the motor cortex [63], and the hippocampus [38, 64]. These alterations in synaptic maturation elicit reductions in neuronal viability [63] and changes in the generation of giant depolarizing potentials, which are the earliest form of forebrain coordinated network activity [61, 65] and whose alterations could impact normal network function later in life [61, 65].

During adulthood, the FKN/CX3CR1 axis is also involved in normal neural function through a variety of actions, including the promotion of adult neurogenesis [66], the integration of new adult-born cells [66–68], the regulation of microglial motility and microglia–neuron interactions [41, 68], neuronal survival [33, 34, 69], neuronal excitability control [70], and, as will be reviewed later, the modulation of synaptic transmission and its plasticity [28, 61, 71]. All these modulations intervene in a variety of brain functions such as the sleep/wake cycle [72], learning and memory [42, 61, 71, 73], mood control [61, 67], food intake control [74, 75], and sensory processing [59, 69].

The role of the FKN/CX3CR1 axis in adult neurogenesis and circuit remodeling in adulthood is reflected in the observation that intracerebral application of exogenous FKN promotes adult neurogenesis [76] and reverses its age-dependent decrease [76]. Moreover, lack of FKN or CX3CR1 blockade reduces adult neurogenesis due to an excess of IL-1𝛽 production [42, 66, 73, 76, 77] and a decrease in microglia–neuron physical contacts [68], which alters the spine density, dynamics, and size of adult-born granule cells in the olfactory bulb [68] and reduces the elimination, formation, and maintenance of synapses on these cells [68]. A similar scenario is observed in adult-born granule cells of the dentate gyrus that exhibit deficient synaptic integration, reduced spine density, and reduced synaptic vesicle availability in the absence of CX3CR1 [67].

CX3CR1 levels change circadianly, with lower levels of CX3CR1 during the light phase and increased levels during the dark phase [72]. The lack of CX3CR1 impacts the sleep/wake cycle and increases the duration of NREM sleep, which correlates with a reduction in hippocampal excitatory neurotransmission [72]. Aside from the circadian changes in the FKN/CX3CR1 axis, other signaling alterations in this system are associated with cognitive functions such as learning and memory [71]. The FKN expression is upregulated in the hippocampus after training in the water maze [71], and researchers have identified alterations in hippocampal-dependent learning and memory, as well as activities of daily living, in CX3CR1-deficient mice [42, 61, 77].

The FKN/CX3CR1 axis also participates in the control of food intake and its disturbance during obesity [74, 75]. Intracerebral administration of FKN reduces food intake, whereas CX3CR1 deficiency increases food consumption [75]. Moreover, FKN reduces obesity and high-fat diet-induced hypothalamic inflammation in these experimental conditions [74, 75].

As mentioned, FKN/CX3CR1 axis activation could produce a neuroprotective environment that is helpful against a variety of insults [33, 34, 78, 79]. However, there is also evidence that activation of the FKN/CX3CR1 axis could promote neuronal damage [80]. Thus, while FKN reduces the neuronal damage induced by 1-methyl-4-phenyl-1,2,3,6-tetrahydropyridine (MPTP) and *α*-synuclein [33, 34], it can also favor 1-methyl-4-phenylpyridinium- (MPP+)-induced neuronal damage, which is prevented by a CX3CR1 neutralizing antibody [80]. Neuroprotective effects of FKN have also been observed against the damage induced by the human immunodeficiency virus (HIV)-associated protein gp120 [4] and glutamate-induced excitotoxicity [79, 81–83], which was dependent on adenosine [79, 81] and the activation of ERK1/2 and PI3K/Akt pathways [82, 83]. FKN can also protect astrocytes against H_2_O_2_-induced oxidative stress and death [84].

### 2.2. Pathological CX3CR1-Dependent Microglial Modulation

Changes in FKN or CX3CR1 expression and their interaction are extremely diverse and occur in a variety of pathological conditions [30, 52, 85–90] and neurological alterations [91–93]. For example, experimental infection with HIV induces the overexpression of both FKN and CX3CR1 in neurons and microglia, respectively but also the expression of CX3CR1 in astrocytes [85]. Spinal cord or spinal nerve injury (Figure 2), along with intraperitoneal administration of LPS, induces the overexpression of FKN and CX3CR1 in neurons and microglia, respectively, without changes in either of them in astrocytes [32, 90, 94, 95]. Thus, clinical and experimental evidence (including the use of CX3CR1-deficient mice) demonstrates that the FKN/CX3CR1 axis is involved in neurological alterations associated with changes in synaptic transmission and its plasticity [67, 96–106], as will be reviewed next.

Alterations in the FKN/CX3CR1 axis play a role in Alzheimer's Disease (AD), a neurodegenerative disorder closely associated with aging, amyloid beta (A*β*) overproduction [97, 98, 107], and alterations in synaptic transmission and its plasticity [97–99, 107]. CX3CR1 levels are enhanced in AD patients and AD transgenic mice [108]. Decreasing these levels alleviates A*β*-induced neuronal damage and amnesia in AD animal models [107, 109, 110]. Regarding FKN, while aging itself has been associated with its decreased expression in animal models [111], FKN levels are altered in a complex manner in AD patients [91–93]. FKN levels increase in the forebrain of middle-stage AD patients [93], whereas they decrease in late stages of AD [91–93]. This is correlated with a reduction of exosomal RNA levels of CX3CR1 [112]. It seems that FKN levels are inversely correlated with the severity of late-stage AD [113]. However, these changes were not observed in the cerebellum or the brainstem at any timepoint [93]. The decrease in FKN levels in the forebrain of late-stage AD patients was also observed in the cerebral cortex and hippocampus of aged AD transgenic mice [114], which is associated with increased A*β* levels [114]. It seems like the FKN/CX3CR1 axis and A*β* bidirectionally interact. For instance, CX3CR1 deficiency induces a reduction in A*β* accumulation in AD transgenic mice [115–117] due to the increased phagocytic activity of microglia [116, 117]; although, opposite effects have been recently reported [107]. On the other hand, A*β* administration leads to an increase in CX3CR1 levels [110] When the CX3CR1 expression is diminished, A*β*-induced microglial activation, synaptic plasticity blockade, and memory impairment are also reduced [110, 115]. In the rTg4510 mouse model of tauopathy, FKN application reduces tau pathology, microgliosis, and memory impairment [118, 119]. The absence of CX3CR1 in humanized Tau transgenic mice exacerbates tau hyperphosphorylation and tau-related pathology [120].

While the levels of CX3CR1 exhibit complex, even opposite, changes in Parkinson Disease (PD) [112, 121], the levels of FKN increase in PD [122], and the ratio of FKN to A*β* has been positively correlated with PD severity and progression [122]. The FKN/CX3CR1 axis influences the pathophysiology of a variety of PD animal models [123], which are mostly based on the neurodegeneration of *substantia nigra* (SN) [123] and are related to an early decreased [124] and a late increased expression of CX3CR1 [80] as well as a late increase in FKN [80]. FKN can reduce microglial activation and neuronal death when the SN is injected with 6-hydroxydopamine (6-OHDA) [123] or MPTP [33], or when alpha-synuclein is overexpressed through a viral vector [34]. In agreement, CX3CR1-deficient mice exhibit more extensive neuronal cell loss when challenged with MPTP or LPS [51], when alpha-synuclein is overexpressed through a viral vector [125] or in PD transgenic mice [126]. However, in the PD animal model induced by MPP+, the preadministration of a CX3CR1 neutralizing antibody suppressed microglial activation, dopaminergic neuron loss, and behavior deficits [80]. Moreover, exogenous FKN promotes microglial activation and dopaminergic neuron degeneration in the SN [80]. Thus, it is likely that differential expression of either FKN or CX3CR1 during the course of the pathological changes induced in the SN could account for the differences among studies [80, 124]. There is extensive evidence that PD-like pathology is closely related to alterations in synaptic transmission and its plasticity [100].

The FKN expression is reduced in the putamen of patients with Huntington's Disease (HD) [101] and in R6/1 HD transgenic mice [101], which has been mechanistically confirmed by the finding that huntingtin reduces the levels of FKN in the StHdh^Q111^ immortalized embryonic striatal cell line [127] and the striatum of R6/2 HD transgenic mice [128]. Moreover, using human postmortem microarrays and network topology analysis, researchers have identified FKN as a gene that is strongly regulated in HD [129]. Reduced FKN expression has also been found in the striatum during aging [130]. Interestingly, FKN administration restored impaired corticostriatal synaptic plasticity in R6/1 HD transgenic mice.

FKN and CX3CR1 levels are increased in epilepsy patients and rodent epilepsy models [102, 131–134]; although, the persistence of such changes in animal models remains controversial [102, 131–134]. It is worth noting that FKN levels can decrease with specific combinations of antiepileptic treatments [135]. Regarding the effects of the FKN/CX3CR1 axis on the hyperexcitability and neurodegeneration associated with epilepsy, FKN is increased in the pilocarpine-induced status epilepticus (SE) rat model [105, 131, 133, 136], contributing to microglial activation, hyperexcitability, and neurodegeneration. These pathological signs are alleviated when the FKN/CX3CR1 pathway is inhibited with a neutralizing antibody or a CX3CR1 antagonist [105, 131, 133, 136] but aggravated with exogenous FKN [131, 136]. Similarly, seizure severity in an epilepsy model induced by kainic acid is exacerbated in CX3CR1^−/−^ mice compared to wild-type animals [47]. Researchers also found a decrease in excitability [137]. Moreover, CX3CR1 absence could prevent neuronal damage but not seizures induced by viral CNS infection [138]. Thus, it is likely that during neurotoxicity and seizures, the effects of FKN/CX3CR1 axis activation could be complex and render both neuroprotective and proepileptic effects [47, 131]. Epilepsy is closely related to changes in synaptic transmission and its plasticity [96, 102–105].

FKN levels are reduced after stroke in patients [139] and after middle cerebral artery occlusion (MCAO) in rodents [140, 141]. However, FKN and CX3CR1 levels have also been found to increase 1 to 14 days after MCAO [30, 142] or even 5 to 8 weeks after bilateral common carotid artery occlusion [143, 144], which could be explained by differences in the ischemic model, the brain area assessed or the time after ischemia induction that samples were collected. These changes in the CX3CR1 expression induced by MCAO have been related to alterations in synaptic pruning, spine density, long-term synaptic plasticity, and cognition [144], all of which can be reversed by multiple mild stimulations (i.e., experiences; [144]). As mentioned, FKN can induce both toxic and neuroprotective effects under ischemic conditions [46]. FKN-induced neuroprotection involves the reduction of caspase-3, NF-*κ*B, and inflammasome activation, which leads to the lessening of infarct size and the attenuation of neurological deficits induced by the MCAO model [46, 141], which is likely triggered by adenosine receptor activation [46]. However, using the same experimental conditions and ischemic model (MCAO) with FKN- or CX3CR1-deficient mice produced a less severe brain injury [46]. Moreover, again, under the same experimental conditions and model, the administration of exogenous FKN increased brain damage in FKN-deficient mice [46], clearly indicating that the protective or toxic effects of FKN depend on the state of the neural circuit microenvironment and/or its concentration. This is important because low FKN levels are associated with severe strokes and higher FKN levels are associated with better patient outcomes regardless of the initial severity of the stroke [140].

These findings coincide with the reduced inflammation, brain injury, and mortality found in other reports after the induction of MCAO in FKN- or CX3CR1-deficient mice [37, 145, 146], which is reproduced by inhibition of the CX3CR1 signaling pathway by a neutralizing antibody [142]. However, it has also been found that CX3CR1 knockdown by small interfering RNA increases the damage induced by the four-vessel occlusion model [147], which could be explained by differences in the ischemic model or the time after ischemia induction that damage was assessed.

The FKN/CX3CR1 axis also plays a part in the regulation of emotional behavior and mood disorders. While a lower CX3CR1 expression has been found in patients with bipolar disorder [148], changes in the connectivity of brain regions related to mood regulation have been observed in mice lacking CX3CR1 [149]. This coincides with increased CX3CR1 levels found in stressed animals [150] and in depressed animals subjected to early-life inflammation [106]. In contrast, depressive-like behavior induced by prenatal stress leads to a decrease in both FKN and CX3CR1 [151]. While FKN reduces inflammation and depressive-like behavior induced by prenatal stress [152], the reduction of CX3CR1 inhibits depressive-like behavior induced by early-life inflammation [106], which involves a reduction in spine alterations due to microglia engulfment [106]. Although some authors did not detect depressive-like behavior in CX3CR1-deficient mice [153, 154], and a few even reported that these mice are resistant to the chronic stress-induced depression [155–157], other authors report that CX3CR1-deficient mice show anxiolytic-like and depressive-like phenotypes in females [67] and greater depressive-like behavior in response to LPS-induced inflammation [158]. The latter is associated with the activation of the indoleamine 2,3-dioxygenase [158] and the former with changes in synaptic connectivity [67]. We have recently corroborated that CX3CR1-deficient mice exhibit signs of anxiety, which is related to changes in synaptic transmission [61]. As mentioned, depression has been associated with changes in synaptic activity and its plasticity [67, 106].

From the variable changes in the FKN/CX3CR axis observed under the pathological conditions just reviewed, which seem to be mediated by TGF-*β* signaling [95], it is fair to state, until this point, that the increase or decrease in CX3CR1 or FKN expression cannot be strictly correlated with neuroprotection or neurotoxicity but, as mentioned before, the observed contradictory effects would depend on the brain area, and/or the pathological conditions in which it is assessed, by mechanisms that will be summarized next.

The FKN/CX3CR1 axis is a strong modulator for the production and release of proinflammatory microglial cytokine synthesis and the microglial response to immunogens [19, 36, 159]. For instance, the activation of microglial cells with LPS allows the synthesis and release of proinflammatory cytokines such as IL-1*β*, TNF-*α*, NO, and IL-6, which were attenuated by FKN treatment [19, 36, 159] (Figures 1 and 2). In addition, the lack of CX3CR1 has been related to enhanced inflammation due to the increased release of proinflammatory cytokines such as TNF-*α*, IL-1*β*, and cC1q [51, 52, 160] (Figures 1 and 2), which correlates with the microglial expression of activation markers including CD45 and MHC II [52]. This microglial activation induced by the absence of CX3CR1 is exacerbated under inflammatory conditions [51, 161–163]. In the diabetic retina, there is an exacerbated subretinal microglial accumulation in CX3CR1^−/−^ mice compared to wild-type animals [161, 163]. In addition, mice lacking CX3CR1 overreact to LPS stimulus with an exacerbated IL-1*β* expression compared to wild-type mice [51, 158, 164]. Besides, damage and seizure severity in a model of kainic acid-induced neurotoxicity is exacerbated in CX3CR1^−/−^ mice compared to wild-type animals [47, 160], suggesting that during neurotoxicity and seizures, the activation of the FKN/CX3CR1 axis could be neuroprotective [47, 160].

The reduction of CX3CR1 signaling can also impact neural function under conditions of mild inflammation [89], such as those found in spontaneously hypertensive rats [89]. In these animals, the CX3CR1 expression is reduced, [89] and microglial morphology is altered, as cells exhibit shorter processes but the same number of endpoints (a proxy of ramifications), in the rostral ventrolateral medulla [89]. These changes indicate that decreased FKN/CX3CR1 axis signaling is related to chronic inflammation and morphological changes in microglial cells toward an activated phenotype [89]. In contrast, there is also evidence that a reduction in CX3CR1 signaling could prevent microglia form exhibiting a proinflammatory phenotype [90, 91, 120]. For instance, in the global cerebral ischemia model, a reduction of CX3CR1 signaling with an anti-FKN antibody reduces neuroinflammation, which is reflected in decreased IL-1*β* and TFN-*α* [165]. In this model, CX3CR1 downregulation protected against demyelination in the striatum, cortex, and hippocampus [90, 165]. Moreover, the specific CX3CR1 inhibitor AZD8797 reduces neuronal apoptosis after spinal cord injury (SCI), along with the expression of IL-1*β*, IL-6, and TNF-*α* [90] (Figure 2). Thus, the neuroprotective effects of FKN could include its antiapoptotic properties in both neurons [15, 85] and microglia [18] and its ability to maintain microglia in an inactivated state [36–39] or diminish their reactivity to toxic stimuli [36–38]. The FKN/CX3CR1 axis is not only involved in the regulation of neural network viability but in its functional and morphological changes, as will be reviewed next.

## 3. Microglial Regulation of Synapsis Structure and Function

Synaptic activity is the ongoing synchronous and asynchronous communication among neurons, determined by action potential neurotransmitter release, stochastic action potential-independent vesicle fusions, and electric coupling [61, 96, 103, 166, 167] Synaptic activity is influenced by pre- and postsynaptic structure and function [61, 96, 103, 166, 167] and is constantly modulated by microglia by regulating spine form and density [168–171], synaptic pruning [56, 172], synaptic remodeling [44, 173–175], synaptic maturation [55, 59, 63], and engulfment of damaged synapses [45, 55, 176].

Functionally, the mere contact of microglial processes with the spines can lead to synaptic synchronization [177]. In addition, microglia can modify synaptic activity by releasing molecules such as IL-33 [173] and IL-1*β* [55]. Alternatively, microglial release of BDNF [169] or IL-10 [178–180] can also modulate spine formation. Moreover, microglia can modulate spine density by releasing TNF-*α* [181, 182] or IL-10 [178].

A variety of microglial receptors participate in sensing the microenvironment and promoting microglia-mediated synaptic modulation [183]. For example, the microglial innate immune receptor TREM2 is required for synapse elimination and normal brain connectivity [183]. Besides, microglia almost exclusively express the purinergic receptor P2RY12 [183–186], whose pharmacological inhibition prevents the neuronal activity-induced synaptic recruitment of microglial protrusions and reduces microglia baseline motility [183], indicating that activity-dependent synaptic ATP release can function as a local chemoattractant that leads to the recruitment of microglia protrusions to active synapses [183]. Once microglial protrusions contact spines, an increase in the frequency of spine calcium transients is observed [177]. This increase is related to the enhancement of synapse activity and the promotion of local network synchronization [177]. In addition, microglia contribute to the structural synaptic changes in response to experience [173, 187], which might depend on IL-33 [173]. Microglia can modulate synapses by releasing not only cytokines but also neurotrophic factors such as BDNF, which is critical in motor learning-induced spine formation [169] and the modulation of spine density and maturity [44, 45, 67, 176].

Microglial depletion is a common tool used to understand the role of microglia in neuronal activity [45, 184, 188–190]. From these experiments, we can summarize that normal microglia activity is required for brain function and plasticity [45, 188–191]. For example, microglia elimination induces neural circuit hyperconnectivity and hyperactivity in adult mouse visual cortex [188], which is related to increases in both excitatory and inhibitory synaptic connections to cortical pyramidal neurons [188]. Microglia depletion exacerbates kainic acid-induced and picrotoxin-induced seizures [192]. However, microglia depletion can also induce the opposite effects since such manipulation can decrease neural synchronization [177], reconfirming that microglial activity and function is closely related to their environmental physiology and the specific experimental conditions of the studied phenomenon. Microglia depletion also reversibly affects AMPA-dependent glutamatergic transmission but not NMDA-dependent glutamatergic transmission [45]. Moreover, glutamatergic synapses, after microglial depletion, exhibit characteristics of immaturity by showing excessive potentiation and defects in synaptic multiplicity, which can be attributed to the fact that the density of dendritic spines is decreased [45]. Behaviorally, when microglia are depleted, mice are unable to discriminate between a novel and a familiar object [45]. All these changes could be reversed by spontaneous microglial repopulation [45]. Interestingly, Elmore et al. [193] showed that the elimination and subsequent repopulation of microglia reverses some alterations in dendritic spine morphology induced by aging.

## 4. Fractalkine/CX3CR1 Regulation of Synapsis Structure and Function

### 4.1. Fractalkine/CX3CR1 Modulation of Synaptic Pruning and Maturation

The exact signals between microglia and neurons used to regulate synaptic activity are not completely understood but clearly include the FKN/CX3CR1 axis [67] (Figures 1 and 2). Since the early embryonic development of neural networks, there is an overproduction of synaptic processes. Some of them are eventually removed by microglia through “synaptic pruning” [56, 106], which continues in mature neural networks [106, 174] and is dependent on CX3CR1 [56, 106]. Thus, microglia synapse engulfment is deteriorated in CX3CR1-deficient mice [58], which has been linked to a higher number of dendritic spines and greater PSD95 immunoreactivity, due to a deficit in synaptic pruning in the developing hippocampus [56, 194] and which correlates with alterations in synaptic transmission [56, 194]. Perhaps because of the alterations in normal neural network wiring, adult mice lacking the CX3CR1 exhibit weak synaptic transmission, lower functional brain connectivity, deficits in social interactions, increases in repetitive behavior phenotypes, and cognitive deficits [39, 42, 44, 61, 175]. A similar scenario can be found upon the absence of FKN [58]. In mice lacking FKN expression, synapse elimination is defective in thalamocortical synapses due to deficient engulfment by microglia [58]. Moreover, Hoshiko et al. [55] demonstrated that mice lacking CX3CR1, due to delayed microglial infiltration, fail to mature their thalamocortical synapses early in development since their AMPAR/NMDAR ratio and their switch from GluN2B to GluN2A NMDA receptor are reduced, in contrast to their increase during normal development [195, 196]. Similarly, Basilico et al. [44] found that CX3CR1^−/−^ mice exhibit immature CA3-CA1 synapses with a reduced AMPA/NMDAR ratio and reduced hippocampal functional connectivity. The increase in the size of hippocampal excitatory postsynaptic currents (sEPSC) in proportion to miniature synaptic currents is also blocked in CX3CR1^−/−^ mice [64], which persists into adulthood [64]. Consequently, in adult CX3CR1^−/−^ mice, CA3–CA1 functional connectivity remains diminished due to a small number of functional synapses between Schaffer collaterals and CA1 pyramidal neurons or to a decrease in their synaptic strength [44, 45]. We have recently found in adult CX3CR1^−/−^ mice that dentate gyrus–CA3 connections remain diminished [61], which is related to changes in microglia morphology, changes in the mossy fiber innervation, and alterations of CA3 functional topology [61], negatively impacting the mood, activities of daily living, and cognition of CX3CR1^−/−^ mice [61].

### 4.2. Fractalkine/CX3CR1 Modulation of Synaptic Transmission

Acute FKN induces a reduction in synaptic transmission reflected as the amplitude of field excitatory postsynaptic potentials (fEPSPs), in a concentration-dependent manner [197, 198], and a reduction in the amplitude of sEPSC through AMPA receptor modulation [4, 83, 176], which is not observed in CX3CR1-deficient mice [176] or in the presence of a CX3CR1 blocking antibody [197]. As mentioned, we have shown that CX3CR1^−/−^ mice exhibit a reduction in the amplitude of sEPSC recorded in CA3 pyramidal neurons along with a reduction in the amplitude of mossy-fiber evoked EPSC [61]. However, some reports indicate no changes in basal synaptic transmission by blocking the action of FKN using a neutralizing antibody [197] or by the absence of CX3CR1 [42, 153, 197]. Adenosine released from microglia is involved in FKN-induced synaptic depression [78, 79, 198]. In fact, FKN-induced sEPSC depression, which is insensitive to pharmacological blockade of both A1R and A2AR, is abolished by the pharmacological blockade of A3R and its knockout [198]. Furthermore, A3R stimulation by selective agonists mimics FKN-induced sEPSC depression [198]. However, it has also been found that FKN increases the amplitude of fEPSPs by enhancing its NMDA-dependent component, which requires the activation of AR2 [199]. Moreover, in the midbrain raphe nuclei, FKN enhances GABA-mediated spontaneous inhibitory postsynaptic current amplitude and increases evoked inhibitory postsynaptic current amplitude in serotoninergic neurons [70]. This effect is blocked by an anti-FKN neutralizing antibody [70].

## 5. Fractalkine/CX3CR1 Modulation of Neuronal Plasticity

### 5.1. Fractalkine/CX3CR1 Modulation of Hippocampal Plasticity

Many studies have focused on the influence of microglia on long-term potentiation (LTP) and long-term depression (LTD) [42, 43, 200–202] (Figure 1). LTP and LTD are forms of long-term neuronal plasticity [99, 203, 204] that consist in a long-term increase or decrease in synaptic efficacy, respectively, that have been closely associated with learning and memory, among other changes in behavioral adaptations [99, 203, 204]. The absence of microglia has been related to differential disturbances in hippocampal LTP [45, 193]. Microglial absence can potentiate LTP in young mice [45], whereas microglial depletion can abolish LTP in aged mice [193]. In both cases, the changes in LTP can be reversed by microglial repopulation [45, 193]. As already mentioned, FKN reduced basal synaptic transmission [176], which is occluded by LTD [197], indicating that both phenomena may share cellular and molecular mechanisms [197]. However, LTD induction is impaired in animals that lack CX3CR1 [153, 197].

FKN administration also inhibits hippocampal LTP (Figure 1) when applied just before and during stimulation [43, 176], an effect that is mediated by A3R [43]. However, as will be reviewed later, FKN may facilitate spinal LTP if added after tetanizing pulses [205]. Alterations in LTP induction have also been tested in CX3CR1^−/−^ animals [42, 45, 153], which are unable to display LTP following high-frequency stimulation [42, 45]. Interestingly, blocking the action of IL-1*β* by administering the antagonist of its receptor (IL-1RA) is sufficient to reestablish this long-term synaptic plasticity [42] (Figure 1). The inability of CX3CR1-deficient mice to achieve LTP correlates with learning deficits [42, 206] that are also reversed by IL-1RA administration [42]. However, there is also evidence that CX3CR1^−/−^ animals have enhanced LTP when induced by a weak stimulation protocol [77], which correlates with better performance in cognitive tests [77]. Differences in the stimulation protocol could account for the differential effects of CX3CR1 absence on LTP induction [42, 77, 116]. FKN-deficient mice show a decrease in LTP [73], which correlates with cognitive deficits and reduced neurogenesis [73]. This effect is reversed by increasing FKN [73]. Despite the described differences, taken together, the results indicate that FKN/CX3CR1 signaling is needed for the normal induction of both LTD and LTP in the hippocampus (Figure 1).

### 5.2. Fractalkine/CX3CR1 Modulation of Spinal Plasticity

High-frequency stimulation-induced LTP at spinal C-fiber synapses has been considered a synaptic model of pathological pain [207] and seems to be modulated by the FKN/CX3CR1 axis [208]. The CX3CR1 expression, which is restricted to microglia in the spinal cord [209], is increased in pain models such as chronic constriction injury (CCI) [32, 209], spinal nerve injury [32, 95], SCI [90], sciatic nerve ligation (SNL) [49, 210], and pain induced by monoarthritis (MA) [211] (Figure 2). After SNL-induced neuropathic pain, the CX3CR1 expression has been found not only in microglia but in neurons and astrocytes [32]. In contrast, FKN, which is exclusively expressed by spinal cord and dorsal root ganglion neurons [32] (Figure 2), does not change after the induction of different chronic pain paradigms such as CCI and SCI [209].

SNL-induced neuropathic pain elicits the release of CatS by microglia [28] (Figure 2), thus promoting the release of FKN by neurons in the spinal dorsal horn [29] (Figure 2). In these pathological conditions, released FKN binds to microglial CX3CR1, leading to a series of cellular responses that include the induction of synaptic facilitation [208] (Figure 2). Among these cellular responses, CX3CR1 activation results in the synthesis and release of proinflammatory cytokines including IL-1*β* [208], which is involved in spinal LTP induction [208] (Figure 2). These findings indicate that the FKN/CX3CR1 axis is relevant in the establishment and/or maintenance of neuropathic pain (Figure 2).

CX3CR1-dependent spinal synaptic facilitation has been proposed as one of the main mechanisms underlying chronic neuropathic pain (reviewed in [212]). For instance, spinal administration of soluble FKN is pronociceptive [49] and induces pain facilitation [48]. In fact, intrathecal FKN administration induces allodynia and thermal hyperalgesia [50], which involves microglial activation [50, 211] and the subsequent release of proinflammatory cytokine synthesis [50, 211].

CX3CR1 activation leads to the activation of the p38 MAP kinase pathway, which is critical for pain development [213] (Figure 2) as it induces the synthesis of proinflammatory cytokines, including IL-1*β*, and subsequently promotes pain [49, 213] (Figure 2). Antagonizing the actions of proinflammatory cytokines decreases exacerbated nociception in rodent models of chronic pain [214]. Particularly, blocking the actions of IL-1*β* through the antagonist of its receptor IL-1RA reduces chronic pain, suggesting that IL-1*β* accumulation triggered by microglial activation induces pain facilitation [208, 215]. Willemen et al. [213] demonstrated that hyperalgesia is dependent on the release of IL-1*β*. They described that the injury signal activates CX3CR1 and subsequently p38 MAPK kinase, culminating in the release of IL-1*β* that worsens pain in mice. Similarly, the induction of spinal facilitation by FKN is independent of TNF-*α* but dependent on IL-1*β*, since nociceptive facilitation is blocked by antagonizing the action of IL-1*β* prior to the addition of FKN [208] (Figure 2).

Reducing CX3CR1 activity by either antagonizing the receptor [48] or using neutralizing anti-CX3CR1 antibodies in an MA model [211] leads to inhibition of pain facilitation. Similarly, in models of allodynia and/or thermal hyperalgesia (neuropathic pain, CCI and spinal nerve injury models), the absence of CX3CR1 activation prevents pain [50], which is related to a reduction in microglia-mediated inflammation [90]. Among other proinflammatory mediators, spinal facilitation depends on the release of NO from activated microglia [48]. Taken together, these data indicate that nociceptive facilitation is dependent on microglial activation by means of CX3CR1, activation of p38 MAP kinase pathways, and the release of proinflammatory cytokines, NO and CatS (Figure 2).

Regarding FKN/CX3CR1 axis involvement in spinal plasticity, the induction sciatic nerve LTP is inhibited with the use of anti-CX3CR1 antibodies in wild-type animals and is not observed in CX3CR1^−/−^ animals [205]. Additionally, this group reported that interleukins mostly released by microglia, such as IL-18 and IL-23, contribute to spinal LTP in a CX3CR1-dependent manner (Figure 2), and the blockade of their actions, with neutralizing antibodies, inhibits spinal LTP [205]. Based on previous data and those of Chen et al. [90] showing that treatment with the CX3CR1 inhibitor AZD8797 prevents microglia from acquiring its proinflammatory phenotype, reducing the expression of proinflammatory cytokines such as IL-*β*, IL-6, and TNF-*α* after SCI and facilitating recovery from spinal cord damage and decreasing pain, we can conclude that the FKN/CX3CR1 axis could be considered a therapeutic target to relieve chronic pain (Figure 2).

## 6. Fractalkine/CX3CR1 Modulation of Respiratory Rhythm Plasticity

A vital function that is highly sensitive to inflammation and microglial modulation is breathing [189, 190, 216, 217], which is generated by the pre-Bötzinger complex (preBötC) [218–220] and can be maintained in a brainstem slice preparation [189, 190, 217, 220, 221]. The preBötC and its motor outputs can adapt their function upon changes in oxygen supply [189, 190, 217, 219–221] and can induce long-term facilitation (LTF) in their activity in response to acute intermittent hypoxia (AIH) [190, 217, 219]. LTF is a form of respiratory plasticity that consists in a long-term increase in inspiratory frequency and/or amplitude evoked by stimulation of respiratory peripheral nerves or AIH [190, 217, 219]. LTF is dependent on both BDNF [222] and adenosine [223]. This plastic change is highly sensitive to microglial modulation [190, 217]. For instance, we have shown that LTF is blocked after microglia depletion and LPS administration *in vitro* [190]. Similarly, systemic LPS administration impairs LTF in a p38 MAP kinase-dependent manner [216]. As already mentioned, p38 MAP kinase is recruited after CX3CR1 activation [213].

We have also found that activating CX3CR1 with FKN induces a depression of the respiratory rhythm generated by the preBötC [189], which is similar to the depression induced by IL-1*β* [224] and which agrees with the observation that systemic application of FKN also reduces breathing *in vivo* [225]. Moreover, the FKN/CX3CR1 axis is involved in breathing alterations induced under pathological conditions [225, 226]. For instance, knocking out CX3CR1 prevents the respiratory alterations observed in a transgenic mouse model of Rett syndrome [226]. In addition, the administration of anti-CX3CR1 monoclonal antibodies prevents the respiratory depression induced by the respiratory syncytial virus [225]. Finally, regarding respiratory plasticity, we have recently found that FKN application prevents the induction of LTF *in vitro* [190], which is reproduced by other microglial modulators [190], indicating that the FKN/CX3CR1 axis modulates breathing generation under normal and pathological conditions but is also required for the proper generation of IH-induced respiratory plasticity.

## 7. Conclusions and Perspectives

In this review, we summarized information on how the CX3CR1 receptor modulates microglial activity and how this modulation is affected by a variety of factors, including the brain structure [42–45], animal model [86, 90, 95], and developmental stage being studied [56]. We also showed that the FKN/CX3CR1 axis modulates the formation, maturation, and presence of spines and synaptic boutons [45, 56, 169, 178], contributing to the remodeling and normal functioning of neuronal circuits to the extent that the absence of this signalling system has been linked to abnormal neuronal activity. We also reviewed that FKN/CX3CR1 signaling modulates different types of plasticity including hippocampal LTP and LTD [42, 44, 45], spinal LTP [205], and respiratory LTF [190], which clearly indicates that the FKN/CX3CR1 axis modulates various brain functions in a state-dependent manner under both normal and pathological conditions. Furthermore, the FKN/CX3CR1 axis is also involved in the modulation of different plastic changes occurring in several neural circuits, which not only reveals the key role of this system in neuron-microglia communication but also indicates that this communication can be targeted to modulate brain function or palliate diverse pathological conditions.

## Abbreviations

HD: Huntington's disease
AD: Alzheimer's disease
AMPA: 2-Amino-3-(5-methyl-3-hydroxyl-1,2-oxazol-4-yl) propanoic acid
NMDAR: N-Methyl-D-aspartate receptor
ATP: Adenosine triphosphate
BDNF: Brain-derived neurotrophic factor
TGF-*β*: Tumor growth factor-beta
CD: Cluster of differentiation
CNS: Central nervous system
CR: Complement receptor
CX3CR1: CX3C motif chemokine receptor 1
IL-1*β*: Interleukin one beta
IL-1ra: IL-1 receptor antagonist
LPS: Lipopolysaccharide
LTP: Long-term potentiation
TNF-*α*: Tumor necrosis factor-alfa
TREM2: Triggering receptor expressed on myeloid cells 2
SCI: Sciatic nerve Injury
SNI: Spinal cord injury
SNL: Sciatic nerve ligation
LTF: Long-term facilitation.
